# The notochord: development, disease and stem cell-based modelling

**DOI:** 10.1242/dev.205337

**Published:** 2026-06-15

**Authors:** Julie Warin, Tiago Rito, James Briscoe, Guillaume Blin, Anne Camus

**Affiliations:** ^1^ Nantes Université, Oniris, INSERM, Regenerative Medicine and Skeleton, RMeS, UMR 1229, F-44000 Nantes, France; ^2^ School of Biomedical Sciences, Li Ka Shing Faculty of Medicine, The University of Hong Kong, Hong Kong SAR, China; ^3^ The Francis Crick Institute, 1 Midland Road, London NW1 1AT, UK; ^4^ Centre for Regenerative Medicine, Institute for Regeneration and Repair, The University of Edinburgh, Edinburgh EH16 4UU, UK; ^5^ Institute for Stem Cell Research, School of Biological Sciences, The University of Edinburgh, Edinburgh EH16 4UU, UK

**Keywords:** Notochord, Stem cell models and organoids, Transcriptomics, Human diseases, Intervertebral disc and spine, Spinal cord

## Abstract

The notochord is a defining feature of chordates. It acts as mechanical support and a source of signals to surrounding tissues during development. In mammals, notochord-derived cells persist within intervertebral discs, where they form the nucleus pulposus, the cartilage in between vertebrae units that provides the spine with flexibility. Here, we synthesise developmental knowledge with recent advances in notochord biology and insights from single-cell molecular approaches. We discuss the developmental processes from notochord initiation during gastrulation through to disc formation, highlighting signalling pathways that govern axial mesoderm specification and notochordal lineage commitment. Knowledge gained from *in vivo* studies has guided the development of pluripotent stem cell-based models in mice and humans, including monolayer and micropatterned systems and 3D organoids. These models recapitulate key developmental aspects of notochord formation and pave the way for disease modelling and regenerative applications. We discuss their relevance to the study of developmental disorders arising from notochord dysfunction and notochordal cell roles in disc homeostasis. Finally, we outline remaining questions and examine how developmental insights and stem cell innovations can advance our understanding of tissue formation, function and homeostasis while fostering the integration of basic mechanistic insights with translational applications.

## Teaser

**Summary:** This Review discusses how developmental and stem cell studies of the notochord can reveal key insights into spine formation and intervertebral disc development and maintenance, providing new platforms for modelling developmental mechanisms and notochord-related disorders in humans.

## Introduction

**Fig. 1.**
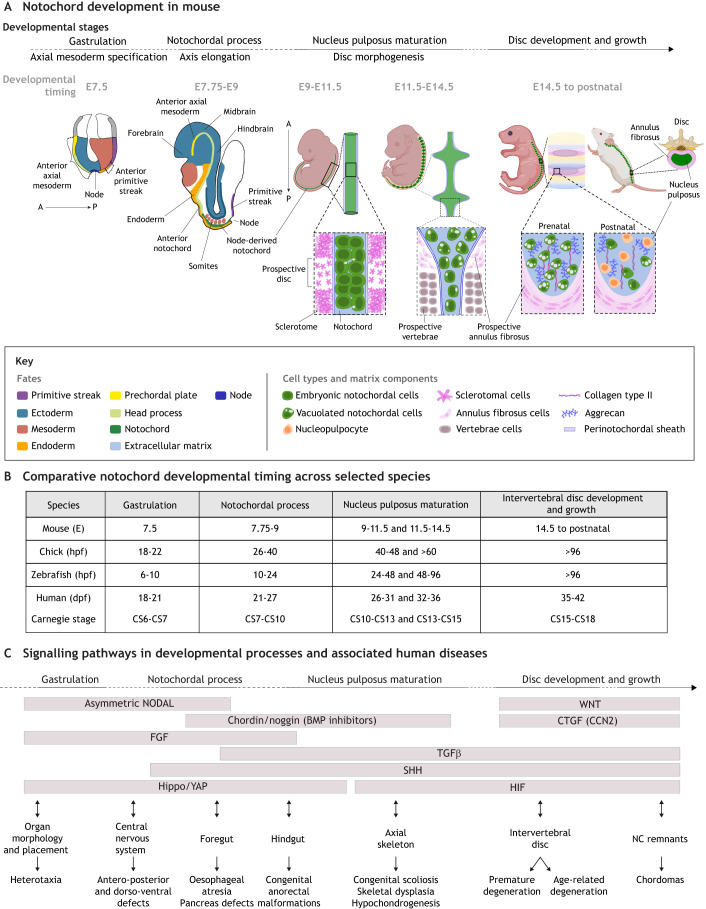
**Overview of notochord development and associated signalling pathways across vertebrate species.** (A) Notochord developmental stages in mice. At the end of gastrulation, the mouse primitive streak extends along the posterior midline, with the node forming at its distal end. Axial mesodermal cells form the head process and the prechordal plate anteriorly, establishing the signalling midline structure. Posteriorly, the notochordal process extends and later separates from the endoderm to generate the definitive notochord. The notochord subsequently undergoes cellular maturation, characterised by vacuolation and extracellular matrix deposition. During vertebral re-segmentation, notochordal tissue is locally lost at the intervertebral regions, while persisting cells within the disc space give rise to the nucleus pulposus. In the postnatal and adult stages, nucleus pulposus cells exhibit morphological and functional specialisation, including differentiation into vNCs and nucleopulposus cells. Anterior-posterior (A-P) axis is indicated. (B) Developmental timing of the notochord in chick, human, mouse and zebrafish. CS, Carnegie stage; dpf, days post-fertilisation; E, embryonic day; hpf, hours post-fertilisation. (C) Signalling pathways involved in vertebrate notochord development and associated human diseases. NODAL initiates asymmetry and midline patterning, while chordin and noggin inhibit BMP signalling. Sonic hedgehog (SHH) secreted by the notochord patterns the neural tube, gut and axial skeleton.

The notochord is a transient rod-like embryological structure that defines chordates. Emerging between embryonic day (E) 7.5 and E9.5 in mice, and between the third and sixth gestational week in humans (de Bree et al., 2018), the notochord runs the rostro-caudal length of the developing embryo, where it performs two distinct roles (Fig. 1A,B). First, the notochord acts as a transient axial skeleton, providing mechanical support before the vertebral column forms in vertebrates. This structural role is accomplished by the fluid-filled vacuolated notochordal cells (vNCs; see Glossary) surrounded by a firm extracellular connective tissue (known as the perinotochordal sheath) creating a rigid but flexible rod. Second, and equally importantly, in all chordates, the notochord acts as a signalling hub providing molecular cues that pattern surrounding tissues during early organogenesis (Fig. 1C). It organises and directs the differentiation of cells in adjacent neural (Placzek and Briscoe, 2018) and mesodermal (Williams et al., 2019) tissue by secreting molecules, notably sonic hedgehog (SHH) and bone morphogenetic protein (BMP) inhibitors such as noggin. In addition to its embryonic roles, in mammals notochord-derived cells persist into the adult where they contribute to the nucleus pulposus, the gelatinous, proteoglycan- and water-rich inner core of the intervertebral discs (Fig. 1A). For most vertebrates, these discs create functional units between vertebral bodies providing compressive resistance, enabling spinal movement and maintaining structural integrity (Fig. 1A). Degeneration of intervertebral discs caused by ageing or injury is a major contributor to chronic lower back pain and disability (Hickman et al., 2022) (Box 1).

Box 1. Notochord-related diseases In addition to their role in developmental processes, NCs are important for intervertebral disc health and function throughout life. Degenerative spine disorders, particularly degenerative disc disease (DDD) resulting from ageing or injury, a condition that has emerged as a major contributor to chronic lower back pain, are a leading cause of disability worldwide (Fig. 1C; Yurube et al., 2023). Notochord-specific deletion of *Ccn2* in mice disrupts intervertebral disc development and accelerates age-related DDD, primarily due to decreased aggrecan and type II collagen production, along with increased type I collagen (Bedore et al., 2013). Regenerative therapies aimed at addressing the primary cause of DDD by targeting NCs and nucleus pulposus cells seek to restore disc structure, function and environment through cellular repair and regeneration. The connection between PSC-based models and their applications in disease modelling and regenerative medicine has prompted increased focus on NCs to address the global burden of DDD, relieve symptoms and restore spinal health for millions of patients.

While most eNCs differentiate to form the nucleus pulposus, remnants remain in approximately 20% of adult vertebrae. These remnant NCs can undergo malignant transformation to form chordomas (Karpathiou et al., 2020), rare cancerous bone tumours that arise primarily at the skull base (clivus for 32%) or lower spine region (sacrococcygeal for 29%). Chordoma cells retain several features of the mature notochord, including a vacuolated cytoplasm (‘physaliphorous’ appearance) while expressing characteristic markers, such as *TBXT* (Halvorsen et al., 2023; Vujovic et al., 2006) and cytokeratins 8, 18 and 19 (Salisbury and Isaacson, 1985). *TBXT* may play a role in chordoma pathogenesis, although this is still debated (D'Agati et al., 2019; Sheppard et al., 2021). Genetic studies have identified *TBXT* gene duplications in familial cases and *TBXT* variants in sporadic tumours. Cyclin-dependent kinase inhibitors disrupt *TBXT* autoregulation, downregulating its expression and inducing apoptosis. Direct *TBXT* degradation induces cellular senescence and sensitises tumour cells to anti-apoptotic inhibitors (Sharifnia et al., 2019).

**Table:** 

**Glossary**
**Annulus fibrosus**
The fibrous outer region of the intervertebral discs, which originates from paraxial mesoderm-derived sclerotome cells. Composed of concentric lamellae of collagen-rich extracellular matrix, it provides tensile strength and structural integrity to the disc, enclosing the nucleus pulposus.
**Anterior mesendoderm**
Tissue derived from the pool of mesoderm and endoderm progenitors arising from the early and mid gastrula organisers prior to node formation. Comprises the prechordal mesoderm and the anterior definitive endoderm contributing to the foregut.
**Arthrotome**
A specialised subdomain of the sclerotome that gives rise to the joints of the spine, including the annulus fibrosus of intervertebral disc anlagen. It contributes to articulation structures by segregating from chondrogenic sclerotome progenitors.
**Axial mesoderm or chordamesoderm**
Midline mesodermal tissue, formed during gastrulation, that gives rise to the prechordal plate, head process and notochord. Positioned below the neural tube, above the gut tube, and flanked by pairs of somites.
**Chondrocyte-like cells or nucleopulpocytes** Specialised nucleus pulposus cells resembling articular chondrocytes in morphology but with only partial transcriptional overlap. They arise from the notochord and progressively expand during ageing and early degenerative conditions. They maintain disc homeostasis by synthesising extracellular matrix components, ensuring hydration and mechanical function.
**Early gastrula organiser**
A transient region of heterogeneous progenitor cells detected in the epiblast of the early-streak embryo that express *Gsc*, *Foxa2*, *Lhx1*, *Eomes*, *Mixl1* and *Noto*. Contributes mainly to non-axial tissues such as cranial mesoderm and heart, collectively termed anterior mesoderm, but also to the prechordal plate, an anterior axial mesoderm derivative.
**Embryonic notochordal cells (eNCs)**
Tightly packed, non-vacuolated cells forming the early rod-like notochord along the anterior-posterior axis of all chordate embryos (including humans). They secrete signalling molecules (e.g. SHH) that pattern surrounding tissues, such as the neural tube and somites, establishing the embryonic midline.
**Head process**
Expresses *Gsc*, chordin, *Otx2*, *Lhx1*, *Dkk1*, *Cer1*, brachyury, *Foxa2*, *Sox9* and corresponds to the anterior-most portion of the notochord, located between the prechordal plate and the node-derived notochord, underlying the prospective mid- and hindbrain.
**Late gastrula organiser or node**
A pit-shaped anatomical landmark forming at the tip of the primitive streak at the end of gastrulation and which persists until the end of axial elongation. The trunk and tail notochord are both derived from the node.
**Mesendoderm progenitor cells**
Population of bipotent cells capable of forming both mesodermal and endodermal derivatives and presumed to be different from defined progenitors of either mesoderm or endoderm because of their ability to respond to specific developmental cues.
**Mid gastrula organiser**
A transient region of heterogeneous progenitor cells detected in the epiblast of the mid-streak embryo that express *Foxa2*, *Gsc*, *Lhx1*, *Eomes*, *Mixl1*, *Noto*, chordin and noggin. This region is composed of early gastrula organiser cells and adjacent anterior epiblast cells, and contributes specifically to the head process, an anterior axial mesoderm derivative, but only minimally to the notochord, whereas adjacent anterior epiblast cells predominantly generate somite and lateral mesoderm. Notochord progenitors are instead found in the anterior primitive streak immediately posterior to the mid gastrula organiser.
**Neuromesodermal progenitors**
*Sox2*^+^ and brachyury progenitor cells that give rise to both neurectoderm and paraxial mesoderm during axis elongation.
**Paraxial mesoderm**
A paired mesodermal tissue flanking the axial mesoderm along the body axis. It segments into somites, which generate the musculoskeletal system, including the dermis, skeletal muscle and most components of the vertebral column.
**Prechordal plate**
Rostral-most portion of the anterior axial mesoderm expressing *Shh*, *Gsc*, *Foxa2*, *Lhx1*, *Dkk1* and *Cer1* that is required for proper forebrain development.
**Sclerotome**
Derived from the ventromedial compartment of each somite, the sclerotome-derived mesenchymal cells give rise to pre-cartilaginous structures as well as connective tissue. Sclerotome cells migrate around the notochord and neural tube and differentiate into the vertebrae, ribs and intervertebral discs.
**Vacuolated notochordal cells (vNCs)**
Large, specialised physaliferous cells with intracellular fluid-filled vacuoles that share features with lysosome-related organelles that arise from eNCs.

Despite the notochord's importance and its clinical relevance to human health, our mechanistic understanding of notochord formation and function has lagged behind other embryonic tissues, such as neural tube, somites or limb buds (Colas and Schoenwolf, 2001; Dubrulle and Pourquié, 2004; Towers and Tickle, 2009). However, the field is experiencing a renaissance driven by several converging advances. Sophisticated molecular and embryological approaches have begun to unravel the complex signalling networks governing notochord development, a topic we explore in depth in this Review. In parallel, the establishment of pluripotent stem cell (PSC)-based models has opened new opportunities to study notochord formation in controlled conditions. Innovative *in vitro* systems will provide mechanistic insights into normal development while offering promising platforms for disease modelling, tissue engineering, and regenerative therapies.

In this Review, we summarise these recent developments, placing them within the broader context of notochord biology, and we highlight crucial questions that remain to be addressed. We also explore how fundamental embryological discoveries, primarily focussing on mouse studies, inform innovative therapeutic approaches, and, conversely, how PSC-based models can enhance our understanding of basic notochord biology.

## Notochord initiation to intervertebral disc formation

**Fig. 2.**
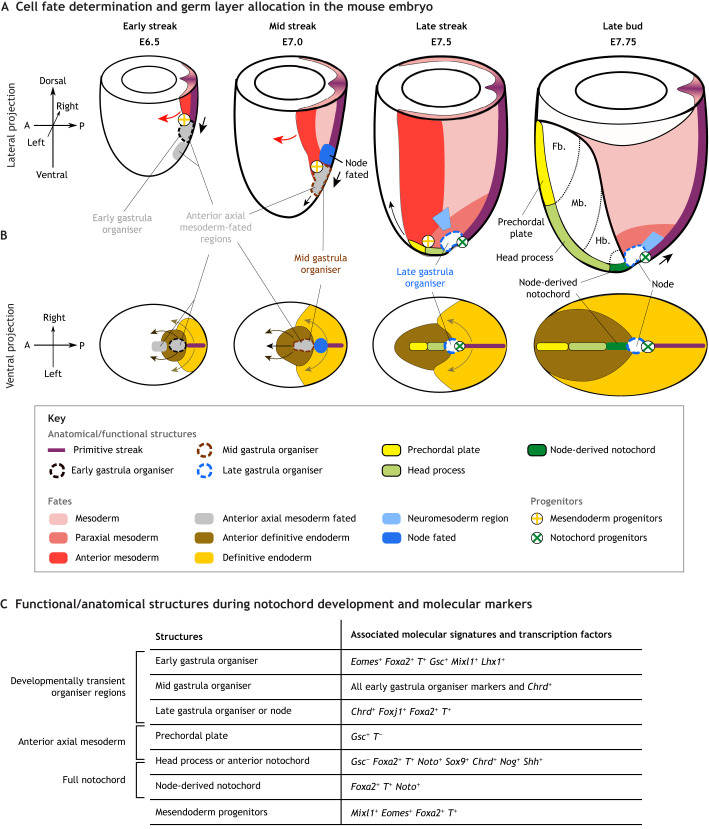
**Developmental origins of the mouse notochord.** (A) Lateral and ventral (B) projections of cell fate and allocation from E6.5 to E7.75 mouse embryo. The definitive endoderm layer is not drawn on the lateral projection to show the progression of primitive streak-derived mesodermal cell fates. The first axial mesoderm-fated cells are found at E6.5 within and anterior to the early gastrula organisers. At this stage, epiblast cells ingressing through the streak migrate anteriorly and generate cranial and cardiac mesoderm and definitive endoderm. As gastrulation proceeds, early gastrula organiser-derived cells will merge with anterior axial mesoderm precursors to form the mid gastrula organiser at E7.0. Anterior mesoderm precursor cells leave the mid gastrula organisers and are displaced anteriorly along the midline to establish the prechordal plate and the head process. The putative location of mesendoderm progenitor cells is shown (yellow cross). At this stage, the node precursors are located posterior to the mid gastrula organisers in the primitive streak. The node becomes apparent at E7.5 with notochord progenitors found in the ventral node–streak border (Wymeersch et al., 2021) while the neuromesoderm-fated region is still found anterior and dorsal to the node (Forlani et al., 2003). As axial elongation begins around E7.75, *Noto*^+^ cells are displaced posteriorly along the crown to generate the trunk notochord. Neuromesodermal progenitors now reside adjacent to notochord progenitors in the caudo-lateral epiblast. The anterior portion of the mesoderm is not drawn at E7.75, to show the location of the prospective forebrain (Fb.), midbrain (Mb.) and hindbrain (Hb.) relative to the prechordal plate and head process. A-P, anterior-posterior body axis. (C) Classical transcription factors/molecular signatures associated with different cell fates during mouse notochord development.

**Fig. 3.**
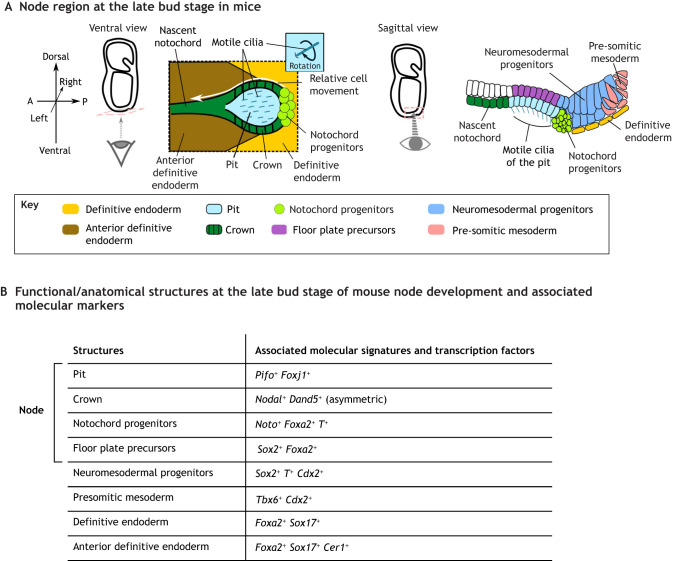
**Anatomy of the node and location of notochord progenitors in E7.75 mouse embryo.** (A) Ventral and sagittal view of the mouse node region. The node appears as a specialised bilaminar structure composed of floor plate precursors in the dorsal layer, and pit cells with motile cilia in the ventral layer flanked by crown cells. The node functions as the left-right organiser (reviewed by Little and Norris, 2021) and forms part of the growth zone, which generates neural, mesodermal and endodermal tissues during axial elongation. A-P axial elongation process is driven by a pool of axial progenitors including neuromesodermal progenitors in close association with the node and notochord progenitors. (B) Functional and anatomical structures with classical associated molecular signatures and key transcription factors. *Pifo* is also known as *Cimap3*.

In amniotes, notochord development begins during gastrulation following the appearance of the primitive streak, a transient embryonic structure that is initiated by the transcription factors brachyury, Mixl1 and Eomes. It arises at the posterior side of the embryo and extends along the midline. The primitive streak is a crucial structure where pluripotent epiblast cells undergo epithelial-to-mesenchymal transition (EMT), enabling cell fate specification and spatial organisation as they migrate through the streak during gastrulation. These processes drive the formation of the three germ layers (ectoderm, mesoderm and endoderm) and establish the embryonic body plan (Lawson et al., 1991). Different regions of the primitive streak give rise to distinct mesodermal subtypes. Lineage-tracing studies in mice have shown that the axial mesoderm has a complex ontogeny with distinct cell populations forming different segments of the notochord along the antero-posterior (A-P) axis in a sequential temporal order (Beddington, 1994; Kinder et al., 2001; Sulik et al., 1994). From early to mid gastrulation, the axial mesoderm arises from small populations of cells that are continuously recruited to specialised organiser regions at the anterior tip of the primitive streak, termed the early gastrula organiser and the mid gastrula organiser (Fig. 2A,B). Pioneer experiments in mice at the single-cell level demonstrated that cells within these early and mid gastrula organiser regions exhibit remarkable fate plasticity, underscoring the pivotal role of immediate environmental cues in regulating the balance between developmental potency and lineage commitment during early embryogenesis (Lawson et al., 1991). In all vertebrates, these organiser regions are complex and dynamic and contain overlapping progenitor zones, which continuously evolve in cellular composition, spatial arrangement and molecular characteristics (reviewed by Tam and Masamsetti, 2025; Kretzschmar et al., 2025; Martinez Arias and Steventon, 2018; Stern, 2025) (Fig. 2C). Both the early and mid gastrula organisers contribute to the anterior axial mesoderm, which comprises the prechordal plate and the head process, both required for proper forebrain development (Filosa et al., 1997; Lewis et al., 2007). At E7.5 in mice, the primitive streak completes its extension with the appearance of the node, which forms the key component of the late gastrula organiser at its distal end (Fig. 3). This marks the end of gastrulation and the onset of A-P axial elongation of the embryo. Evidence from transplantations and lineage tracing in chick and mouse embryos suggest that the node contains resident stem cells and acts as an instructive niche imparting a notochord or somitic fate to the cells transiting through it during the early stages of axial elongation (reviewed by Solovieva et al., 2022; Wymeersch et al., 2021). In vertebrates, while the early and mid gastrula organisers form the anterior axial mesoderm, the node serves as the primary source of the trunk and tail segments of the notochord during axial elongation.

**Fig. 4.**
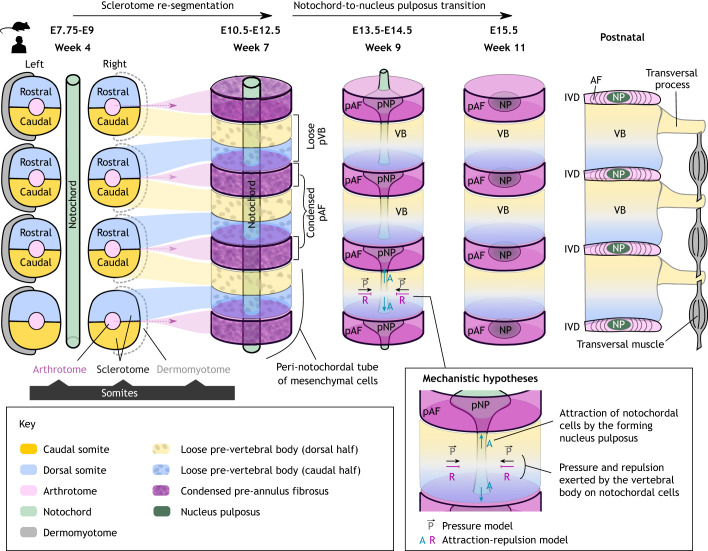
**Sclerotome re-segmentation and notochord-to-nucleus pulposus transition in mice and humans.** During axis elongation, pairs of somites are formed flanking the notochord. The somites are polarised from their inception with the presence of a rostral (blue) and a caudal (yellow) half each defined by a distinct gene expression profile. Both halves are epithelial and undergo epithelial-to-mesenchymal transition (EMT) to give rise to the sclerotome and the dermomyotome. The somites also enclose a pool of mesenchymal cells called the somitocoele forming the arthrotome (pink), which gives rise to the annulus fibrosus. Re-segmentation refers to the fact that there is no direct correspondence between a somite and a future vertebra. Instead, the sclerotome undergoes a reorganisation whereby the caudal half of one somite recombines with the rostral half of the adjacent somite to create the prospective vertebral bodies, illustrated here with shaded areas of yellow and blue. The dermomyotome remains in its original relative position to connect adjacent vertebrae (transversal muscle). The sclerotome- and arthrotome-derived mesenchymal cells invade the perinotochordal space as they differentiate and reorganise into alternating loose and condensed domains along the rosto-caudal axis. Subsequently, zones of loose and condensed mesenchymal cells become apparent in the perinotochordal tube (E10.5-E12.5; Week 7), to give rise to prospective annulus fibrosus and vertebral body, respectively, ensuring proper segmentation and alignment of the vertebral column. In the mouse embryo at E13.5-E14.5 (Week 9), two non-exclusive mechanistic hypotheses have been proposed for the process of notochord segmentation or involution underlying the formation of the prospective nucleus pulposus, represented in the bottom-right box, with the pressure model (in grey), and the attraction-repulsion model in blue and red. The notochord ultimately differentiates into the nucleus pulposus, which becomes positioned at the centre of the annulus fibrosus (from E15.5; Week 11). Developmental stages are indicated in embryonic days for mice and in weeks post-conception for humans. AF, annulus fibrosus; IVD, intervertebral disc; pAF, pre-annulus fibrosus; pNP, pre-nucleus pulposus; pVB, pre-vertebral body; VB, vertebral body.

The distinct cellular ontogenies translate into distinct morphogenetic mechanisms: cells of the head process and node-derived trunk notochordal cells (NCs) (starting approximately at the third or fourth somite in mouse) intercalate into the definitive endoderm to establish the notochordal plate at the embryonic midline through medio-lateral convergent extension movements, in mice, chicks and humans but not in zebrafish. By E9.0, these mesodermal cells separate from the endoderm to form the definitive notochord (McDole et al., 2018; reviewed by Balmer et al., 2016; Lee and Anderson, 2008). In contrast, the tail notochord (posterior to the hindlimb region at E10.5 in mouse) arises from a distinct subpopulation of *Nodal*-expressing peripheral (crown) node cells that actively migrate toward the posterior of the elongating embryo (Yamanaka et al., 2007). Despite this developmental complexity, the mature notochord emerges as a single, continuous rod-shaped structure expressing *Foxa2*, brachyury, *Sox9*, *Shh*, chordin and noggin (Barrionuevo et al., 2006) along its entire A-P length, enabling coordinated signalling function throughout the developing embryo. As embryonic development continues, the notochord is crucial to the patterning of the adjacent neural tube and paraxial mesoderm-derived somites while undergoing its own morphogenesis (Fig. 1A). This ultimately leads to the formation of the segmented vertebral column and the conversion of the notochord into the specialised tissue within intervertebral discs. The transformation begins with SHH signalling from the notochord promoting the differentiation and proliferation of ventral regions of the somites into sclerotomes and their subsequent migration and segmentation, a crucial step in axial skeleton formation, conserved in all vertebrates (Chiang et al., 1996). Through a re-segmentation process, the mechanism of which remains debated in the field, sclerotome cells reorganise along the A-P axis into alternating condensed and loose regions, giving rise to the annulus fibrosus of the intervertebral discs and to the vertebral bodies, respectively, which later form the segmented vertebral column (Draga and Scaal, 2024) (Fig. 4).

The mouse embryonic notochord initially appears as a uniform rod-like structure composed of tightly packed cells within the perinotochordal sheath, an acellular basement membrane rich in laminin and collagens (Götz et al., 1995) that separates the notochord from adjacent tissues. Concurrent with the surrounding tissue changes, the notochord itself exhibits maturation and morphological transformation (Warin et al., 2026). The notochord undergoes differentiation, during which it acquires characteristic physaliferous vacuoles, large fluid-filled intracellular structures that define its vacuolated morphology, before subsequent reorganisation (Hunter et al., 2004). In regions corresponding to the developing vertebral bodies, the notochord regresses completely through a process also described as notochord involution. In contrast, in the intervening regions, where the condensed sclerotome forms the annulus fibrosus, the mature vNCs become concentrated and specialised to form the nucleus pulposus. Evidence in the chick indicates that the sclerotome provides patterning cues to the notochord and regulates its segmentation, ensuring that both tissues develop in register with one another (Ward et al., 2018). During zebrafish notochord segmentation, the sheath differentiates into alternating cartilage-like and mineralising domains, providing a template for spine patterning (Lleras Forero et al., 2018; Wopat et al., 2018). Although documented in other model organisms, such as chick and zebrafish, this segmentation process remains speculative in the mouse (Fig. 4). Since no gene has yet been shown to exhibit a segmental expression pattern in the notochord, it has been proposed that the notochord segmentation results instead from physical forces exerted by the surrounding sclerotomes (Lawson and Harfe, 2015). The result is the formation of the intervertebral discs, the fibrocartilaginous structures that ensure load-bearing capacity and resistance to deformation throughout life. The highly hydrated nucleus pulposus, derived from the transformed notochord structure, serves as the shock-absorbing core of the disc and is prone to degenerative changes in mammals (Hickman et al., 2022). Given its pivotal role in disc formation, the morphogenetic mechanisms guiding the notochord maturation and transformation into the nucleus pulposus, as well as how this process is orchestrated during vertebral body formation, warrants further investigation.

## Emergence and segregation of the notochordal lineage

### Transcriptional regulation

The literature on the transcriptional regulation of the notochord spans decades of research in various chordate model organisms from invertebrate chordates, such as amphioxus and ascidians, to birds, amphibians, fish and mammals (reviewed by Di Gregorio, 2020). This has highlighted an evolutionarily conserved battery of transcription factors involved in notochord formation. In particular, the forkhead box transcription factor FOXA2 (also known as hepatocyte nuclear factor 3, beta) and the T-box transcription factor brachyury (encoded by *TBXT* in humans; known as *T* in mice) lie at the core of the notochord gene regulatory network. In the notochord, *Foxa2* and brachyury comprise a positive cross-regulatory interaction and activate the transcriptional cascade that imparts and maintains the notochord identity (Lolas et al., 2014; Tamplin et al., 2008). While a core gene regulatory network is conserved, the selective pressure imposed by the diverse environmental conditions in which the embryos of different species develop has led to gene duplications and alterations of the cis-regulatory modules that drive tissue specificity, expression levels and timing of various notochordal developmental genes (Yasuoka, 2020). In consequence, it makes the direct cross-species comparisons of transcriptional programmes difficult (Lolas et al., 2014; Nishizaki et al., 2001). Adding to this complexity, genetic analyses in mice have demonstrated differential regional requirements for key transcription factors: while FOXA2 is essential for the specification of all axial mesoderm segments (Ang and Rossant, 1994; Weinstein et al., 1994; Yamanaka et al., 2007), brachyury is dispensable for the formation of anterior axial mesoderm that comprises prechordal plate and head process (Herrmann, 1995).

Regional expression of developmental genes along the A-P length of the notochord in ascidians (Oda-Ishii and Di Gregorio, 2007; Reeves et al., 2021) and amphioxus (Albuixech-Crespo et al., 2017) suggests that differential gene patterning is a conserved trait that likely emerged early in chordate evolution. In mice, one such gene, the homeobox gene *Noto*, which is specifically expressed in the node and posterior notochord, represses its own transcription in the anterior notochord and head process (Abdelkhalek et al., 2004; Alten et al., 2012; Beckers et al., 2007). Although loss of *Noto* results in only moderate morphological defects in the node and posterior notochord, live-imaging and lineage-tracing studies have shown its pivotal role in maintaining the identity of node-derived NCs in mice (Yamanaka et al., 2007). Indeed, in the absence of *Noto*, mutant axial mesoderm cells mislocalise into the paraxial mesoderm and downregulate brachyury and *Foxa2* expression, showing that regional expression of notochord genes such as *Noto* have implications in cell fate decisions or maintenance. The vast majority of notochordal genes identified so far (Fig. 2C) are maintained throughout the length of the notochord in mammals and it remains to be determined whether regional transcriptional differences exist in the notochord of birds and amphibians. Nevertheless, in species such as ascidians where regionalisation exists, subtle differences in the expression profiles of notochordal genes could be associated with the diverse ontogenies of the cells forming the notochord. In ascidians, the notochord arises from two distinct lineages under the influence of distinct signalling pathways (Di Gregorio, 2020). This raises the question of how notochord-defining *Foxa2* and brachyury expression is controlled in axial mesoderm cells with distinct developmental trajectories in vertebrates (Fig. 2). The list of notochord-specific cis-regulatory modules is growing (Jeong and Epstein, 2003; Kemmler et al., 2023; Negrón-Piñeiro et al., 2024; Nishizaki et al., 2001; Schifferl et al., 2021, 2023; Tamplin et al., 2011) due to integrated approaches and greater accessibility to *in vitro* models. Further analyses of these cis-regulatory modules should help resolve the question of *Foxa2* and brachyury regulation under different developmental contexts. For example, motif analysis of the node and nascent notochord proximal enhancer of mouse *Noto* has revealed binding motifs for transcriptional effectors of key signalling pathways, such as TCF/LEF1 (WNT signalling) and TEA domain (TEAD) proteins (involved in Hippo signalling) (Alten et al., 2012), consistent with findings that WNT and Hippo signalling cooperate to maintain notochord identity. This raises the question of what signalling pathways instruct cells to become notochord and how these pathways are integrated to initiate the notochord transcriptional programme.

### Signalling pathways

In mice, the signalling pathways active during gastrulation, including NODAL, BMP, WNT, fibroblast growth factor (FGF) and NOTCH, have all been implicated in the specification of the notochord. Although important insights are highlighted below, the precise signalling requirements for each segment of the mouse notochord remains to be clarified.

#### NODAL

NODAL signalling is essential for axial mesoderm specification. Genetic perturbations of the NODAL pathway in mouse embryos have highlighted that the anterior axial mesoderm is more sensitive to NODAL signalling perturbation than the node and posterior notochord (Chu et al., 2004; Episkopou et al., 2001; Robertson, 2014; Vincent et al., 2003). This observation has suggested that specification of the anterior axial mesoderm requires higher NODAL signalling. Alternatively, differentially expressed intracellular factors may confer distinct sensitivities to the NODAL pathway, which is the case for ZIC2, a member of a conserved zinc finger family of transcription factors that physically interacts with SMAD2 and SMAD3 (intracellular mediators for TGFβ/NODAL signalling) to control *Foxa2* expression. This regulatory mechanism has been demonstrated in both mouse and *Xenopus* models (Houtmeyers et al., 2016). Although three ZIC family members (ZIC2, ZIC3 and ZIC5) are co-expressed across most of the gastrulating embryo, potentially providing functional redundancy, only *Zic2* is expressed within the mid gastrula organisers where the head process originates (Elms et al., 2004). This specific expression profile might explain the greater vulnerability to NODAL pathway disruption in the specification of axial mesoderm anterior to the node. Because crosstalk within the anterior axial mesoderm has been reported, with prechordal plate identity depending on signals from the head process, its loss under mild *Nodal* perturbation may result indirectly from mis-patterning of the head process (Camus et al., 2000; Warr et al., 2008). In contrast to NODAL, BMP signalling inhibits anterior axial mesoderm fates in *Xenopus* embryos (Yasuo and Lemaire, 2001). Mice lacking both noggin and chordin, two inhibitors of the BMP pathway, fail to form the prechordal plate and head process, while the node and posterior notochord persist in the double-mutant embryos (Bachiller et al., 2000). This phenotype could also result from compensatory mechanisms involving other BMP inhibitors, expressed in the node region. For example, BAMBI, a pseudo-receptor inhibiting BMP and NODAL signalling (Onichtchouk et al., 1999), has recently been shown to be expressed adjacent to the chick and mouse node region (Rito et al., 2025).

#### WNT signalling

The WNT/β-catenin signalling pathway triggers the genetic cascade that defines the notochordal lineage in all chordates (Di Gregorio, 2020). In mouse, *Foxa2*, brachyury and *Noto* are direct targets of the WNT pathway, with WNT-responsive regulatory elements present in both brachyury and *Noto* enhancers (Alten et al., 2012; Kemmler et al., 2023; Schifferl et al., 2021, 2023; Schwaiger et al., 2022). While WNT signalling is necessary for anterior axial mesoderm specification and notochord maintenance in the mouse (Di Gregorio, 2020; Lickert et al., 2005; Ukita et al., 2009), in *Xenopus* anterior axial mesoderm specification requires WNT inhibition (Yasuo and Lemaire, 2001). The apparent interspecies differences might be reconciled by considering the timing and axial position at which experimental evidence was observed. In mice, the requirement for WNT signalling is clearly established for the posterior notochord. In addition, the finding that its targets brachyury and *Noto* are dispensable for prechordal plate and head process formation suggests that WNT signalling may not be required for their specification. However, modulation of WNT activity in anterior morphogenesis is essential, with its perturbation leading to anterior axial mesoderm defects (Costello et al., 2015; Ip et al., 2014; Lewis et al., 2007, 2008). Taken together, the precise contribution of WNT signalling to the development of all axial mesoderm derivatives remains to be fully resolved.

#### NOTCH

NOTCH signalling regulates the early development of the axial mesoderm. The complex spatial pattern of NOTCH signalling components in and around the node (Przemeck et al., 2003) is required for the function of the node as the left/right asymmetry organiser (Kato, 2011). Forced activation of NOTCH in the whole epiblast induces a complete absence of anterior axial mesoderm structures in mice, but this phenotype may be secondary to the attenuation of NODAL signalling in these embryos (Souilhol et al., 2015). However, in *Xenopus* and chick, elevation of NOTCH signalling in the organiser expands the floor plate at the expense of the notochord (Gray and Dale, 2010; López et al., 2003). Specifically, Latimer and Appel (2006) demonstrated in zebrafish that NOTCH plays two distinct roles: it both specifies a subset of midline precursors in the shield as the hypochord (an anamniote-specific structure) at the expense of the notochord and promotes the proliferation of floor plate cells. Thus, the NOTCH pathway appears to regulate the allocation and proliferation of progenitor cells between the notochord and the floor plate lineages emerging from the node, although this mechanism remains to be confirmed in mammals.

#### FGF and Hippo signalling

FGF signalling through the activation of MEK protein kinases has been implicated in the induction of brachyury expression and notochord development in several species including ascidians (Amaya et al., 1991; Fletcher and Harland, 2008; Kim and Nishida, 2001; LaBonne and Whitman, 1994; Yamaguchi et al., 1994), although its precise role remains to be resolved. Mechanical forces and cell–matrix interaction (Pulina et al., 2014) also have an influence on anterior axial mesoderm specification and maintenance, although these remain largely underexplored. Recent evidence demonstrates that the activation of the Hippo signalling effector Yes-associated protein (YAP), a key mechanosensor and mechanotransducer, induces *Foxa2* expression in the notochord. Together YAP and FOXA2 synergistically induce *Shh* expression, suggesting that YAP regulates both notochord formation and subsequent neural tube patterning (Cheng et al., 2023). During gastrulation and axis elongation, tissues undergo morphogenetic movements, such as convergent extension, invagination, and cell intercalation resulting in extensive tissue deformations that impose physical constraints on cells. The interplay between physical constraints and signalling cues from organiser regions is a conserved feature of chordate gastrulation (Imuta et al., 2014; reviewed by Wright and Wilson, 2025). Many questions remain on how these mechanical forces influence the specification of the axial mesoderm and the formation and maintenance of the notochord across both temporal and spatial dimensions.

## Mechanisms of notochord maintenance and intervertebral disc formation

### Molecular cues

In mice, fate-mapping studies using *Shh-Cre* and *Noto-Cre* transgenic lines have demonstrated that all nucleus pulposus cells derive from embryonic NCs (eNCs), resolving longstanding uncertainties regarding their cellular origin (Choi et al., 2008; McCann et al., 2012). The transformation of the embryonic notochord into the nucleus pulposus begins around E10.5 in mice with the formation of the sheath and occurs concurrently with the migration of the sclerotome-derived mesenchymal cells (Christ and Wilting, 1992; Paavola et al., 1980). During this period, NCs begin developing their characteristic vacuolated morphology (Hunter et al., 2004). At E14.5, large vNCs, which are markedly distinct from the eNCs, have expanded at the level of the future disc to shape the newly formed nucleus pulposus (Figs 1 and 4). In contrast, by E15.5 the forming vertebral body region contains only remnants of the basal lamina, with NCs fully regressed. However, the molecular signals coordinating notochord maturation and nucleus pulposus formation remain poorly defined, particularly in the context of concurrent vertebrae development (Lawson and Harfe, 2015). A recent study using 5-ethynyl-2′-deoxyuridine (EdU) injection to map proliferation showed that the enlargement of the nucleus pulposus involves localised changes in notochord proliferation, prior to hypertrophic differentiation of the vertebral body region in mice (Long et al., 2025). In line with this observation, another study reports that the balance of the proliferative capacity of NCs and programmed cell death within condensed sclerotome cells, together with matrix remodelling and vacuolation dynamics, drive notochord-to-nucleus pulposus morphogenesis along the rostro-caudal axis in mice (Warin et al., 2026). Deciphering how localised signalling regulates distinct proliferative and apoptosis dynamics of NCs in mammals remains an open challenge (Levillain et al., 2021; Ward et al., 2017, 2018). The core notochordal transcription factors FOXA2, brachyury, NOTO and SOX9, which maintain notochordal identity, stay active throughout the process. However, few studies report that significant transcriptional changes occur during nucleus pulposus formation in mice and humans (Peck et al., 2017; Rodrigues-Pinto et al., 2016). Indeed, comparative gene expression analysis between mouse E12.5 NCs and newborn nucleus pulposus cells reveals differential expression of over 5000 genes (Peck et al., 2017). Notably, *Shh* expression decreases in newly formed nucleus pulposus compared to eNCs, while TGFβ, insulin-like growth factors 1 and 2 (IGF1, IGF2) and extracellular matrix (ECM) components become upregulated in the formed nucleus pulposus. Together, these results highlight that the transition from the notochord to the nucleus pulposus involves significant cellular, molecular and structural changes (Smits and Lefebvre, 2003). However, the specific regulatory programmes (i.e. key transcription factors, signalling pathways and epigenetic modifications) that govern this transition are not fully characterised.

### Biophysical cues

In addition to molecular signals, mechanical forces exerted by the developing vertebral body play a role in the redistribution of NCs within the forming vertebrae and the future intervertebral regions (Jurand, 1974). In vertebrates, at present two mechanistic hypotheses have been proposed: the pressure model claims that compressive forces from vertebrae formation drive NC displacement, while the attraction-repulsion model claims that molecular gradients guide NC localisation (Fig. 4; Lawson and Harfe, 2015; Wang et al., 2011). Supporting the first model, removal of SHH from the mouse notochord (before E10.5) prevents vertebrae formation, eliminating the putative ‘squeezing’ force that moves NCs into intervertebral spaces. This results in a persistent rod-like notochord structure without nucleus pulposus formation. Similarly, mutations altering *Pax1* expression in the mouse sclerotome lead to persistence of the notochord within the vertebral body region associated with increased NC proliferation (Rodrigo et al., 2003; Wallin et al., 1994). This strongly suggests that sclerotomes may signal back to the notochord to control its expansion. In addition, the integrity of the sheath that physically constrains the NCs is essential for their survival and proper nucleus pulposus morphogenesis (Choi and Harfe, 2011; Corallo et al., 2013; Smits and Lefebvre, 2003). The importance of the notochord–ECM interaction was further highlighted in conditional β1 integrin knockout mice, which displayed discontinuous notochord due to impaired convergent-extension movement that is controlled by non-canonical WNT/planar cell polarity. These mice also exhibited intervertebral disc and spine defects, such as misaligned, malformed vertebral bodies and truncated tails (Guo et al., 2020). The importance of the crosstalk between sclerotomes and NCs during nucleus pulposus morphogenesis is also suggested in a notochord-specific Jun kinase knockout study. Mutant mouse embryos display vertebral fusions due to increased NC apoptosis during nucleus pulposus formation (Behrens et al., 2003). Moreover, Hippo pathway activity also contributes to spine development, with TEAD1, TEAD2 and the co-activator YAP being essential for notochord maintenance in mice, likely controlling proliferation and apoptosis (Sawada et al., 2008).

The presence of intracellular fluid-filled vacuoles in the developing notochord provides hydrostatic pressure and this mechanical role is crucial for notochord elongation and stiffness during axis elongation in zebrafish (Ellis et al., 2013; Wang et al., 2017). There is indeed a well-documented correlation between a disorganised extracellular sheath, impaired vacuolation and the regulation of apoptosis during notochord development and axis elongation in vertebrate model organisms (Malikova et al., 2007; Parsons et al., 2002; Wopat et al., 2018). There is also evidence that these vacuoles may be a limiting factor for cell proliferation, suggesting that NCs prioritise vacuole inflation and mechanical function over rapid division (Hong et al., 2019). Once the nucleus pulposus is formed, vNCs help maintain hydration and ECM homeostasis, and their loss or dysfunction is linked to premature intervertebral disc degeneration in mammals (Bach et al., 2016; Merceron et al., 2014; Smolders et al., 2013). Crucial to the developmental timing of the notochord-to-nucleus pulposus transition is the progressive establishment of a specialised ECM within the nucleus pulposus structure in vertebrates (Hwang et al., 2014). The ECM components serve dual roles: they provide biomechanical support for compressive force resistance and create a regulatory environment for growth factor binding and distribution. Two key components, type II collagen (COL2) and aggrecan (ACAN), contribute to the mechanical function of the ECM. COL2 is essential for the notochord-to-nucleus pulposus transformation, as demonstrated by *Col2a1*-deficient mice, which retain rod-like notochord structures until birth and fail to form intervertebral discs (Aszódi et al., 1998). Additional matrix components, including COL6 and small leucine-rich repeat proteoglycans (BGN, FMOD, PRELP), also show increased expression as the nucleus pulposus forms. They play important roles in ECM assembly by binding to collagen fibrils and regulating growth factors such as TGFβ and IGF1, whereas COL6 may contribute to cell mechanotransduction through its pericellular localisation, as generally shown in mouse articular cartilage (Zelenski et al., 2015). Consistent with the biomechanical role of the notochord during axis elongation and vertebral development, defects in notochord formation can cause skeletal abnormalities. In zebrafish, fragmented vacuoles reduce notochord stiffness, resulting in vertebral malformations and progressive spinal curvature (Bagwell et al., 2020; Gray et al., 2014). Similarly, mutations disrupting vacuole biogenesis, such as those affecting *Dstyk* (which codes a dual serine/threonine and tyrosine protein kinase that functions as a cell death regulator), cause asymmetric notochord compression during vertebral bone formation (Fig. 1C; Sun et al., 2020).

The transformation of the notochord into the nucleus pulposus is governed by a complex interplay between molecular and biophysical cues, which together ensure the precise timing and spatial organisation required for notochord regression, nucleus pulposus maturation and the establishment of a mechanically competent disc. Further insights are needed to fully elucidate the regulatory networks and biomechanical interactions that orchestrate cellular differentiation and tissue morphogenesis.

## Disc postnatal development, ageing and degeneration

Postnatal development in mammals coincides with changes in ECM composition and another crucial cellular transition: large vNCs are progressively replaced by an intermediate cell type, the juvenile nucleus pulposus cells, which display reduced vacuolation, high metabolic activity and are key regulators of ECM production and disc growth. In turn, these juvenile nucleus pulposus cells gradually disappear and are replaced by smaller chondrocyte-like cells, also called nucleopulpocytes (Fig. 1). These cellular phenotype shifts start from 22 weeks post-conception in humans and continue primarily during the first decade of life, representing a pivotal change in disc biology before skeletal maturity (Hunter et al., 2004). The transition from a vNC to a vacuole-free, chondrocyte-like cell phenotype, accompanied by decreased proteoglycan content and diminished regenerative potential, is believed to contribute to disc degeneration. N-cadherin (CDH2), a cell–cell adhesion marker, is found highly expressed by vNCs and their direct transitional progeny, the juvenile nucleus pulposus cells, but is progressively lost with ageing. CDH2, through its interaction with β-catenin signalling, preserves the characteristic of healthy juvenile nucleus pulposus phenotype by maintaining cellular morphology, proteoglycan synthesis and the ability to form cell clusters (Hwang et al., 2016). During postnatal life, the nucleus pulposus environment becomes increasingly challenging, characterised by hypoxic, hypertonic and avascular conditions (Urban et al., 2004). *In vitro* disc-relevant biophysical cues, such as hyperosmolarity, have been shown to reduce *CDH2* expression while increasing the expression of the gene encoding the gap-junction protein connexin-43 (also known as *GJA1*), thereby altering mechano-transductive signalling and promoting cellular differentiation toward chondrocyte-like cell phenotype associated with ageing (Palacio-Mancheno et al., 2018). The *HIF1A* gene is a key regulator of the cellular response to hypoxia in intervertebral discs (Tran et al., 2013). In mice, targeted deletion of *Hif1a* specifically in the embryonic notochord results in cell death and premature disappearance of vNCs and derivatives just after birth (Merceron et al., 2014). Moreover, natural ageing alters the disc environment, leading to the loss of NCs and their protective functions, which is central to the degenerative process (Erwin et al., 2006, 2011; McCann and Séguin, 2016). Species such as mice, rats, pigs, bovines and non-chondrodystrophoid dogs that retain higher numbers of vNCs are less susceptible to degenerative disc disease (DDD; Box 1). This correlation highlights the importance of maintaining NC populations for disc health throughout life (Bach et al., 2022; Bruggeman et al., 2012). In humans, whether the nucleus pulposus fully derives from the notochord remains to be formally established as systematic studies and RNA-sequencing, single-cell atlases of nucleus pulposus samples have revealed marked cellular heterogeneity. These studies describe diverse cell populations, including notochordal-like cells and chondrocyte-like cells, fibrocartilaginous phenotypes, subpopulations of progenitor-like cells and stress- or inflammation-associated cells, with the relative abundance of these subpopulations shifting with age and in response to degeneration (Gan et al., 2021; Jiang et al., 2022; Richardson et al., 2017; Tam et al., 2020; Tan et al., 2024; Zhang et al., 2024a). These transcriptomic studies also provide valuable insights for expanding the repertoire of lineage-specific markers, enabling precise tracking of phenotypic changes over time.

### NC homeostasis and regeneration

The importance of NCs for the formation and maintenance of intervertebral discs throughout life has focussed attention on their potential use for regenerative purposes. The homeostasis of nucleus pulposus within intervertebral discs involves a balance between anabolic (ECM synthesis) and catabolic (ECM degradation) activities. This determines matrix turnover, ECM hydration, cellular phenotype and composition, biochemical conditions (oxygen and nutrient supply) and mechanical properties essential for physiological loading. NCs regulate this homeostatic balance through two primary mechanisms. First, they have the capacity to synthesise ECM components, in particular proteoglycan, to maintain the high water content that gives the nucleus pulposus its shock-absorbing properties. Second, and equally important, they function as paracrine signalling centres, secreting trophic factors that support the viability and function of neighbouring nucleus pulposus cells within the disc environment. The molecular mediators of this paracrine function include proteins such as TGFβ, CTGF (CCN2), SHH, WNT and SPARC (Bach et al., 2017; Bian et al., 2017; Huang et al., 2010; Kennon et al., 2018; Lu et al., 2017; Mehrkens et al., 2017; Rajesh and Dahia, 2018; Winkler et al., 2014; Zhu et al., 2025), which have been characterised in exosomes and ECM components derived from NCs and healthy nucleus pulposus tissue across multiple species, including humans (Schmitz et al., 2022). The presence of these signalling molecules within the nucleus pulposus environment emphasises the crucial role of intercellular communication in maintaining tissue integrity. Although many questions remain concerning how these signalling molecules exert their effects, they represent potential targets for therapeutic intervention. Further investigation into these homeostasis mediators, which exhibit pro-anabolic, anti-inflammatory, anti-angiogenic and anti-cell death functions on nucleus pulposus cells, may reveal their capacity to drive disease-modifying effects and regeneration, offering promising avenues for intervertebral disc repair strategies (Bach et al., 2019; Piazza et al., 2020; Tsingas et al., 2020).

The clinical potential of replacing NCs in ageing intervertebral discs to counteract DDD (Box 1) is tempered by major technical hurdles, particularly regarding their isolation, expansion and long-term maintenance. Two principal methodologies are widely used for culturing native NCs, each with distinct limitations (Williams et al., 2023). Three-dimensional (3D) alginate bead culture better preserves the NC phenotype but severely limits cell adhesion and proliferation, lacks physiologically relevant ECM remodelling and mechanical properties, and impairs cell recovery for downstream analyses and translational applications. By contrast, two-dimensional (2D) monolayer culture allows easier experimental control and handling but rapidly promotes cell de-differentiation, compromising the characteristics that make NCs therapeutically valuable. Recent consensus guidelines emphasise that culture parameters, including medium composition and osmolarity, substrate stiffness, oxygen tension and mechanical loading, crucially influence NC phenotype maintenance (Williams et al., 2023). Several promising alternative approaches are being developed to overcome these limitations. For instance, explant-based models maintain the native nucleus pulposus microenvironment and some systems enable vNC expansion through subculturing while preserving more of their native characteristics (Paillat et al., 2023). Biomaterial approaches using hydrogels grafted with bioactive peptides or decellularised matrix scaffolds show potential for overcoming culture limitations by mimicking the native microenvironment required to sustain NC activity and the same applies to nucleus pulposus cells (Barcellona et al., 2020; Basatvat et al., 2023; Laagland et al., 2025). Perhaps the most promising approach is induced PSC (iPSC) technology (Colombier et al., 2020; Liu et al., 2014; Tang et al., 2018; Tong et al., 2024); recent advances in generating notochordal-like cells from human iPSCs have demonstrated their differentiation into matrix-producing cells that partially rescue degenerative phenotypes when transplanted in animal models (Sheyn et al., 2019; Zhang et al., 2020).

Clinical translation of this notochord-based research will require validation in relevant DDD preclinical models that accurately mimic human pathology (Box 1), alongside consistent cellular outcome assessments, including cell survival, integration, ECM production and restoration of disc structure and function within living organisms (Binch et al., 2021). In the light of recent clinical trials, the choice between different therapeutic cell types, particularly mesenchymal stem cells versus iPSC-derived NCs, remains an active area of investigation (Hickman et al., 2022; Pers et al., 2024). While mesenchymal stem cells are widely used for their chondrogenic capacity (Clarke et al., 2014; Colombier et al., 2016), they lack developmental relevance to nucleus pulposus tissue and display considerable patient-to-patient variability. These limitations emphasise the importance of exploring alternative, developmentally aligned cell sources with rejuvenating potential (Bach et al., 2022; Clouet et al., 2019; Rodrigues-Pinto et al., 2018). Therefore, fundamental insights into NC biology, fate and phenotypes are essential for advancing regenerative strategies. The integration of developmental insights with regenerative medicine approaches offers unprecedented opportunities to address key open questions in notochord development, as well as model notochord-related human conditions such as DDD (Box 1) and other spine defects.

### The notochord in disease

Given its essential roles in both structural support and signalling, disruptions to notochord development or function trigger a broad range of medical conditions (Fig. 1C). For instance, dysregulation of SHH signalling activity, mutations in the *Shh* gene or in other genes such as *Zic2* and *Col2a1* (in particular the alternative splice variant procollagen IIA) that influence SHH signalling (Hamdi-Rozé et al., 2020; Leung et al., 2010; Roessler et al., 1996; Warr et al., 2008), can result in holoprosencephaly (Aoto et al., 2009; Barratt et al., 2022; Cohen, 1989; Dubourg et al., 2018), which manifests as a spectrum of brain malformations ranging from complete forebrain fusion (alobar) to partial separation (lobar).

Signalling from the notochord also influences development of the intestinal tract. In pancreatic development, the notochord represses, rather than activates, *Shh* in dorsal endodermal cells. This repression occurs through notochord-secreted activin and FGF2, which promote the expression of pancreatic-specifying transcription factors *PDX1* and *ISL1* (Hebrok et al., 1998). Aberrant notochord signalling in this context contributes to pancreatic developmental disorders, although further work is needed to characterise these potential pathologies.

Defects in the formation of the notochord and its separation from the dorsal endoderm are linked to oesophageal atresia with tracheoesophageal fistula (Fausett et al., 2014; Li et al., 2007). In mice lacking the BMP inhibitor noggin or treated with the chemotherapeutic Adriamycin, the notochord remains tethered to the endoderm (Hajduk et al., 2012). This results in the incorporation of dorsal foregut cells into the notochord, leading to foregut hypocellularity and branching of the notochord. Genetic reduction of *Bmp7* in noggin-null embryos rescues both the notochord defects and oesophageal atresia (Fausett et al., 2014).

Given the role of the notochord in axis elongation and vertebral development, defects in its formation can lead to skeletal abnormalities (Kemmler et al., 2023; Pennimpede et al., 2012; Schifferl et al., 2021; Zhu et al., 2016) in mice, zebrafish and humans, particularly congenital scoliosis-like spinal deformities (Sun et al., 2020). In addition, defects in cilia assembly or function lead to ciliopathies, pleiotropic diseases that may present with abnormal skeletal development and scoliosis (Oliazadeh et al., 2017; Wise et al., 2020). In humans, *TBXT* variants are associated with sacral agenesis (underdevelopment of the sacrum), persistent notochordal canal and abnormal ossification of the vertebral bodies (Postma et al., 2014). A *COL2A1* variant has been also associated with a persistent notochord and abnormal vertebral development with hypomineralisation with failure to form intervertebral discs (Codsi et al., 2015).

Beyond developmental disorders, NCs are important for intervertebral disc health and function throughout life. However, persistence of NC remnants in the adult vertebrae also appears to be the origin of malignant tumours (Al Shihabi et al., 2022; Choi et al., 2008) (Box 1).

The integration of developmental insights with regenerative medicine approaches offers unprecedented opportunities to address key open questions in notochord development, as well as in notochord-related human conditions. Moreover, understanding the roles of the notochord in disease pathogenesis offers potential therapeutic targets for developmental disorders, skeletal deformities, degenerative conditions and notochord-derived malignancies. Further research into notochord biology offers promising avenues for relevant human disease modelling and may reveal novel approaches to treating these diverse but interconnected conditions.

## *In vitro* models of notochord and nucleus pulposus formation

Disc cellularity decreases progressively from birth, with further marked loss in nucleus pulposus cells during ageing and degeneration (Liebscher et al., 2011). Owing to the poor intrinsic repair capacity of the disc and limited accessibility to native human NCs and nucleus pulposus cells, direct studies remain challenging. To overcome these biological, technical and ethical barriers, strategies have been designed to differentiate PSCs into NCs or nucleus pulposus-like cells for *in vitro* modelling. These approaches, often informed by knowledge gained from embryology and developmental biology studies, aim to recapitulate key aspects of the *in vivo* signalling cues, such as the activation of WNT and NODAL/activin/TGFβ signalling pathways. Pioneering works by Winzi et al. (2011) and Xu et al. (2021) have demonstrated the *in vitro* generation of notochord progenitor cells from mouse PSCs, laying a foundational framework but also emphasising the main obstacles and key challenges, including limitations in cell sorting, uncertainties about the precise identity of the cells over time in culture and the difficulties in directing spatial organisation of the developing tissues and controlling relevant patterning of surrounding cells.

**Fig. 5.**
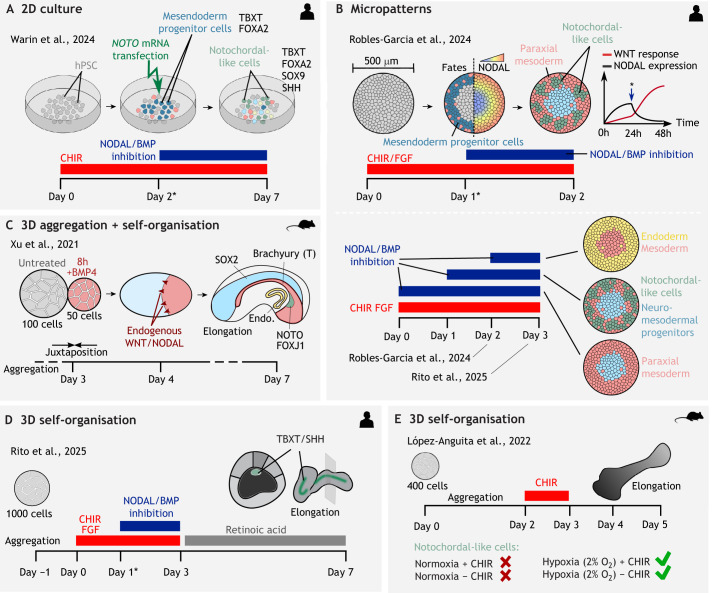
***In vitro* models of notochord development.** (A-E) Several studies have now reported the generation of notochordal-like cells from mouse and human PSCs, using a variety of approaches summarised here in diagrammatic form. Each grey panel focuses on a specific approach: 2D monolayer (A; Warin et al., 2024), micropatterns (B; Robles-Garcia et al., 2024; Rito et al., 2025), 3D aggregation (C; Xu et al., 2021) and self-organisation (D,E; Rito et al., 2025; López-Anguita et al., 2022). A reference to illustrate each approach and whether human or mouse cells were used are indicated. Each panel contains a high-level overview of the protocol used by the authors, with the timing and duration of biochemical treatments shown above a timeline expressed in days. The cell fate outcome and the resulting spatial organisation are also shown. Asterisks indicate the timing of TGFβ inhibition to emphasise the requirement for delayed TGFβ inhibition in multiple protocols. On micropatterns and in 3D models, a self-organised network of endogenous signalling takes place downstream of biochemical treatment, shown with red arrowheads at day 4 in C and with a colour code on half of the micropattern colony at day 1 in B. WNT/NODAL antagonism was identified on micropatterns whereby NODAL inhibition potentiates the cells responsiveness to WNT signalling, indicated in the corresponding line plot in B. Findings on micropatterns highlight that the timing of TGFβ inhibition determines different cell fate outcomes. 3D aggregation with delayed TGFβ inhibition induces a notochord-like rod structure (D). 3D aggregation in the presence of hypoxia promotes the emergence of notochordal-like cells, although their location inside the organoid was not documented (E). CHIR, CHIR99021; Endo., endoderm.

*In vitro* systems that replicate the evolving NC niche by mimicking the dynamic microenvironment supporting NC survival, function and potency to differentiate into nucleus pulposus cells are essential for the generation of large quantities of bona fide NCs required for biochemical and translational studies. Such systems also hold promise for the development of organoids containing notochord tissue, enabling the study of neural and somitic dorso-ventral patterning and related developmental processes (Fig. 5).

### 2D monolayer models

Comparing monolayer-based strategies (Colombier et al., 2020; Diaz-Hernandez et al., 2020; Liu et al., 2014; Warin et al., 2024; Winzi et al., 2011; Zhang et al., 2020, 2024b) reveals that a common essential step is the initial induction of mesendoderm progenitor cells. These cells are precursors of the notochord but also retain the potential to differentiate into definitive endoderm and other non-axial mesoderm derivatives (Scheibner et al., 2021; Kinder et al., 2001; Lawson et al., 1991; Tada et al., 2005). Lineage tracing *in vivo* (Masamsetti et al., 2025) and spatiotemporal transcriptomic analyses of mouse embryos (Peng et al., 2019; Yang et al., 2026; Probst et al., 2021) support the existence at gastrulation stage of *T^+^*, *Foxa2^+^*, *Eomes*^+^, *Mixl1^+^*, *Mesp1^+^* mesendoderm progenitor cells that can form anterior mesoderm, anterior axial mesoderm and anterior definitive endoderm (Fig. 2). *In vitro*, mesendoderm progenitor cells are typically generated through the activation of WNT signalling combined with NODAL/activin/TGFβ pathways, recapitulating the early patterning signals of the primitive streak (Funa et al., 2015; Lam et al., 2014; Loh et al., 2014; Tsankov et al., 2015). Interestingly, recent single-cell studies combining transcriptomic and chromatin accessibility profiling suggest that FOXA2 and EOMES initially pioneer chromatin accessibility in response to WNT and NODAL, priming cells to respond to subsequent signalling inputs for fate consolidation (Yang et al., 2026). Guiding PSCs through the developmental step of mesendoderm progenitor cells significantly enhances the efficiency and fidelity of downstream notochordal differentiation. Starting from mesendoderm progenitor cells, notochordal-like cells can be derived using stepwise protocols with specific signalling cues (Colombier et al., 2020; Warin et al., 2024; Zhang et al., 2020). Zhang et al. (2020) achieved this through NODAL/activin/TGFβ and WNT activation combined with BMP and retinoic acid inhibition, while Colombier et al. (2020) combined similar activations with transient *NOTO* mRNA transfection, thereby demonstrating the role of NOTO in coordinating the gene network underlying human notochord differentiation. Warin et al. (2024) refined the process with a monolayer-based protocol involving BMP and NODAL/activin/TGFβ inhibition (Fig. 5A).

### 2D micropattern models

Another approach uses spatially constrained 2D colonies of PSCs as the starting condition to standardise geometry, confinement and cell density. This improves reproducibility by restricting each colony to limited cell fates and offers a trade-off between complexity and tractability (Blin, 2021). Colonies, typically circular, are generated by micropatterning of ECM substrates and have been used successfully to model aspects of gastrulation or trunk formation with the addition of BMP4 or canonical WNT signalling (Martyn et al., 2018, 2019; Morgani et al., 2018; Ortiz-Salazar et al., 2024 preprint; Warmflash et al., 2014). These model systems have proven particularly useful to gain insights into the signalling dynamic underpinning cell fate decisions (Heemskerk and Warmflash, 2016). Micropattern *in vitro* models containing human notochordal-like cells have now been developed using FGF and Chiron (CHIR99021) as the differentiation triggers to mimic the environment of the human primitive streak (Rito et al., 2025; Robles-Garcia et al., 2024) (Fig. 5B). Analyses of the signalling events downstream of WNT stimulation in micropattern colonies have shown a complex relationship between WNT levels and the downstream dynamics of endogenous TGFβ signalling (Camacho-Aguilar et al., 2024; Robles-Garcia et al., 2024). These complex signalling dynamics guide the differentiation of various primitive streak-associated cell fates in micropattern colonies. Importantly, notochord specification requires transient endogenous NODAL signalling driving the specification of mesendoderm progenitor cells, followed by an abrupt inhibition of the pathway to direct notochord at the expense of endoderm specification (Robles-Garcia et al., 2024; Rito et al., 2025). The need for precise timing of TGFβ inhibition might explain why *in vitro* generation of notochord-like cells previously remained elusive. The recent *in vitro* findings raise the question of how precise signalling is regulated in the complex and dynamic environment of an embryo. We speculate that a plausible explanation is that the timing of cell ingression in organiser regions defines the signalling cues experienced by the cells (Fig. 2). In the future, live imaging with faithful signalling reporters could help to elucidate the question of how signalling dynamics is controlled to trigger cell differentiation at a very precise time and place in the embryo.

### 3D models

Similar signalling protocols are exploited in 3D models of early trunk formation and to generate organoids featuring a notochord cell population (Fig. 5C-E). These models consist of a notochord-like structure surrounded by a paraxial mesoderm population, with both being enclosed by a neural epithelium (Rito et al., 2025). As in the embryo, the number of notochordal-like cells is not high; however, an ECM-rich sheath is observed, along with progressive ventral patterning by SHH (Fig. 5D). Examining the differences in induction efficiency across distinct 3D architectures, alongside aligning *in vitro*-derived progenitors with their *in vivo* counterparts, can guide future strategies to improve the generation of notochord progenitor cells within different *in vitro* systems (López-Anguita et al., 2022; Fig. 5E), including gastruloids (Hamazaki et al., 2024), axioloids (Yamanaka et al., 2023), somitoids (Miao et al., 2023), segmentoids (Miao et al., 2023) and trunk-like 3D structures (Bolondi et al., 2024). Spatial positioning and mechanical cues are crucial for notochord development within an organoid, where the notochord typically forms adjacent to the neural tube and somites (Rito et al., 2025). Beyond minimal models for patterning, there is a need for reproducible architecture to study signalling cues and mechanical forces that shape organoids and understand cell movements and interactions that guide morphogenesis.

The growing availability of single-cell transcriptomic data from human embryonic tissues (Tyser et al., 2021; Warin et al., 2024; Xiao et al., 2024; Xu et al., 2023; Yuan et al., 2025) now makes comparisons of notochord molecular signatures with those of *in vitro* differentiations possible (Rito et al., 2025; Warin et al., 2024; Yaman and Ramanathan, 2023; Box 2), underscoring both the strengths and limitations of stem cell-derived notochord models. They recapitulate early transcriptional states and key features of notochord identity and signalling, yet they fail to fully capture later differentiation and maturation associated with ECM remodelling observed *in vivo*. Correlation analyses and the identification of stage-specific signatures (Box 2) reveal the relevance of currently used *in vitro* systems to study different developmental aspects of notochordal origin and lineage.

Box 2. Molecular signatures: native notochord and stem cell-based models Comparative analysis of transcriptomic data from human embryonic tissues (Tyser et al., 2021; Warin et al., 2024; Xu et al., 2023; Yuan et al., 2025; Zhou et al., 2023) with *in vitro* NCs derived from PSCs (Rito et al., 2025; Warin et al., 2024; Yaman and Ramanathan, 2023) offers valuable insights and perspectives on NC modelling. Following dimensionality reduction, single-cell transcriptomic data analyses show similarity between *in vitro*-obtained cells and their *in vivo* counterparts across a panel of 1488 human genes. Principal component analysis showed that NCs segregated primarily by developmental stage. Correlation analyses confirmed relationships and alignment of *in vitro* models with embryonic stages. The findings resulting from this analysis, including described datasets, have been desposited in Figshare (https://doi.org/10.6084/m9.figshare.31521748).

Differential expression analysis indicated that ‘early-stage’ models and Carnegie stage 7-9 samples retain node/late gastrula organiser-like features, including transient neuromesodermal progenitor signatures, whereas ‘late-stage’ models express the cartilage-related markers *SOX5/6/9* and ECM genes, consistent with early structural maturation of NCs. *ACAN* was under-expressed in *in vitro* cell clusters compared with *in vivo* samples, indicating incomplete maturation across all *in vitro* datasets analysed. Nevertheless, the circadian clock gene *ARNTL* (also known as *BMAL1*) was expressed in early-stage models, suggesting a potential role for circadian regulation in the differentiation and maturation of notochordal-like cells. This aligns with its established functions in development and ECM remodelling, as previously described *in vivo* (Dudek et al., 2023; Gonçalves et al., 2012; Umemura et al., 2022). Pathway-level analyses highlighted dynamic signalling activity, underscoring the role of the notochord as a signalling hub.

The ability to generate NCs from PSCs thus represents a significant opportunity, given the scarcity of embryonic notochord tissue in humans and even in model organisms. Stage-specific signatures emphasise the value of these *in vitro* systems for disease modelling: early-stage NCs, enriched in node-associated genes, such as *PIFO* (*CIMAP3*), show potential to model mechanisms associated with ciliopathies, while late-stage NCs with elevated *SHH* provide a prospective platform to model midline signalling disorders such as holoprosencephaly.

Progressively, single-cell RNA sequencing and spatial omics are building comprehensive biomarker panels that enable the delineation of distinct stages of NC differentiation and maturation, essential tools for monitoring the progression of iPSC-derived notochordal-like cells into specialised, ECM-secreting cells that resemble healthy juvenile nucleus pulposus cells (Gan et al., 2021; Zhang et al., 2024a). These technologies are also crucial for modelling ageing and degenerative changes *in vitro*, enabling a better understanding of NC-driven rejuvenation mechanisms in pathological contexts. The development of robust *in vitro* models, including 2D and 3D culture systems, organotypic disc cultures, and hydrogel-based platforms, enables detailed study of cell–cell and cell–matrix interactions, as well as secretory profiles and cellular behaviours, under controlled conditions.

## Open questions and concluding remarks

The notochord, once regarded as a transient embryonic structure, is emerging as key to understanding development, regeneration and disease. The advent of PSC-derived 2D and 3D models, together with high-throughput transcriptomics, has created opportunities to revisit classical developmental paradigms and interrogate lineage progression and cell fate choice in humans. These developmental processes have remained largely inaccessible to direct investigation due to technical and ethical constraints. Comprehensive molecular atlases of human and model organisms refine the current knowledge of the axial mesoderm molecular profile defined across developmental stages and enable standardised classification of *in vivo* and *in vitro* NC-related populations. Transcriptomic approaches not only allow us to assess how *in vitro* models recapitulate developmental programmes, but also reveal human-specific features of notochord biology. They provide insights into cell fate specification, lineage trajectories and developmental timing at single-cell resolution. These datasets are also indispensable for identifying novel regulators and context-specific signalling combinations that govern notochord emergence and fate. These advances now allow us to map the notochord in new detail and to uncover its various roles during development and beyond.

*In vivo*, NC identity is acquired in a dynamic yet spatially and temporally controlled signalling and mechanical environment. Recent work highlights the interplay between several signalling pathways as central to axial mesoderm specification. *In vitro* findings also confirm that cell fate patterning is influenced by WNT and NODAL signalling; while these pathways cooperatively induce mesendodermal identity, they also antagonise each other's intracellular activities in mice and humans. It can be hypothesised that dynamic Hippo signalling regulates successive stages of axial mesoderm specification and maintenance *in vitro* through coordinated modulation of YAP activity, WNT and FGF/ERK signalling. Moreover, the timing of TGFβ inhibition is emerging as a crucial determinant of notochord induction *in vitro* (Fig. 5). Altogether, these findings bring us closer to understanding the minimal molecular requirements for NCs to emerge *in vitro* but also prompt important questions, such as how this induction occurs in the embryo, and whether the principles uncovered *in vitro* are also relevant *in vivo*. 3D live imaging combined with cell tracking and lineage reconstruction using brachyury and *Foxa2* reporter lines (Imuta et al., 2013) has enabled the generation of a dynamic single-cell reference atlas of mouse development from gastrulation to early organogenesis (McDole et al., 2018). This resource represents a unique opportunity to trace the origin, fate and behaviour of descendants of the node and notochord, as well as those of the lateral and paraxial mesoderm. A systematic exploration of cell specification, lineage segregation and tissue interactions during these crucial stages will establish a framework for in-depth investigations of the underlying molecular mechanisms.

A transcriptomic atlas of human notochord development as well as greater knowledge of lineage-specific epigenetic patterns is important to benchmark how faithfully *in vitro* systems recapitulate developmental trajectories and to determine what type of axial mesoderm cells are generated in culture. Together, *in vivo* studies from all model organisms reveal a remarkable dynamic process for axial mesoderm specification, in which cells transiently pass through signalling centres (early and mid gastrula organisers and node) before acquiring their final position, identity and function (Kretzschmar et al., 2025; Simpson et al., 2024).

Integrating *in vivo* and *in vitro* findings, the emerging model proposes that WNT and NODAL signalling first cooperatively induce *FOXA2* and *TBXT* to establish mesendodermal competence in vertebrates. The timing of axial mesoderm emergence during gastrulation influences the specific signalling environment each cell is exposed to and subsequently their final contribution to the prechordal plate, head process, and anterior or posterior notochord. While significant progress has been made, crucial challenges remain. In particular, the dynamic and specialised niches involved in notochord development have yet to be distinctively recapitulated *in vitro.* Moreover, a complete cranio-caudal axial mesoderm has yet to be simultaneously generated within an organoid system. Furthermore, very little is known about the patterning mechanisms that distinguish pit cells from crown cells in the ventral node, or about how some progenitors in the node–streak border eventually engage in contributing to the node-derived notochord. The close proximity of the two distinct and not intermixed progenitors – notochord progenitors and neuromesodermal progenitors – that drive axial elongation across vertebrates raises the question of which cellular and molecular mechanisms maintain this specific pattern arrangement throughout embryonic development. NODAL signalling may play a regulatory role (Forlani et al., 2003), and by maintaining an epithelial structure, neuromesodermal progenitors may be protected from NODAL stimulation, which usually prompts mesendodermal differentiation and loss of axial progenitor identity (Fig. 2) (Etoc et al., 2016; Legier et al., 2023; Zhang et al., 2019). *In vitro* models may help elucidate the underlying molecular mechanisms and reveal insights into these cell fate regulatory processes.

Future iterations of *in vitro* models, incorporating micropatterned systems, self-organised 3D multicellular organoids or assembloid models, advanced imaging, molecular tools and computational modelling will enable deeper investigation into notochord functions. This includes roles in tissue patterning and morphogenesis, particularly the underexplored regulatory crosstalk between notochord and sclerotomes, which likely influences vertebrae and disc formation and orchestrates skeletal development (Aszódi et al., 1998; Warin et al., 2026). Coupled with biomechanical studies of sheath formation and notochord regression, these insights may reveal how developmental dynamics set the stage for long-term tissue homeostasis. These insights will also guide the development of more refined model systems best suited for specific biological questions or translational applications. Key challenges remain, including improving differentiation efficiency, stabilising cellular identity, controlling cell–cell interactions and tissue architecture, and promoting tissue maturation toward organ primordium. In particular, better insights are needed into the matrisome, cell–cell and cell–matrix interactions, and the role of vacuolation and perionotochordal sheath in notochordal biology.

The evolutionary diversification and biomechanical adaptations of the notochord remain poorly understood. Cross-species studies of notochord emergence and the signalling and mechanical roles of the notochord could uncover conserved transcription factors and cis-regulators, novel pathways, and the evolutionary innovations that enabled the notochord to diversify its structural and signalling roles (Negrón-Piñeiro et al., 2024; Raghavan et al., 2023).

Finally, recent protocols demonstrate that PSCs can be guided toward notochordal or nucleus pulposus-like lineages, representing a promising translational avenue for disc degeneration. Production of notochordal-like cells will allow further investigation into their biology and NC-associated secreted regulatory molecules, paving the way for the characterisation of essential players for healthy disc maintenance. The choice of a biologically relevant and well-characterised cell source is crucial for the success of cell-based therapies. Understanding how epigenetic regulators and ECM cues promote notochordal maturation will contribute to optimisation required to ensure the functional competence of therapeutic cells for regenerative medicine in the musculoskeletal field. As *in vitro* systems improve, they will also become platforms for addressing fundamental developmental biology questions as well as for modelling diseases arising from defects in notochord structure or function, including holoprosencephaly, spinal deformities, DDD and chordoma. Together, these evolving tools are transforming the notochord field, bridging developmental biology with next-generation human disease models and providing a foundation for regenerative medicine.
